# Genome-Wide Association Analyses Highlight the Potential for Different Genetic Mechanisms for Litter Size Among Sheep Breeds

**DOI:** 10.3389/fgene.2018.00118

**Published:** 2018-04-10

**Authors:** Song-Song Xu, Lei Gao, Xing-Long Xie, Yan-Ling Ren, Zhi-Qiang Shen, Feng Wang, Min Shen, Emma Eyϸórsdóttir, Jón H. Hallsson, Tatyana Kiseleva, Juha Kantanen, Meng-Hua Li

**Affiliations:** ^1^CAS Key Laboratory of Animal Ecology and Conservation Biology, Institute of Zoology, Chinese Academy of Sciences (CAS), Beijing, China; ^2^College of Life Sciences, University of Chinese Academy of Sciences, Beijing, China; ^3^Institute of Animal Husbandry and Veterinary Medicine, Xinjiang Academy of Agricultural and Reclamation Science, Shihezi, China; ^4^State Key Laboratory of Sheep Genetic Improvement and Healthy Breeding, Xinjiang Academy of Agricultural and Reclamation Science, Shihezi, China; ^5^Shandong Binzhou Academy of Animal Science and Veterinary Medicine Academy, Binzhou, China; ^6^Institute of Sheep and Goat Science, Nanjing Agricultural University, Nanjing, China; ^7^Faculty of Natural Resources and Environmental Sciences, Agricultural University of Iceland, Borgarnes, Iceland; ^8^All-Russian Research Institute of Genetics and Farm Animal Breeding, Russian Academy of Sciences, Moscow, Russia; ^9^Production Systems, Natural Resources Institute Finland, Jokioinen, Finland

**Keywords:** sheep, prolificacy, genome-wide association study, biological pathways, regulation

## Abstract

Reproduction is an important trait in sheep breeding as well as in other livestock. However, despite its importance the genetic mechanisms of litter size in domestic sheep (*Ovis aries*) are still poorly understood. To explore genetic mechanisms underlying the variation in litter size, we conducted multiple independent genome-wide association studies in five sheep breeds of high prolificacy (Wadi, Hu, Icelandic, Finnsheep, and Romanov) and one low prolificacy (Texel) using the Ovine Infinium HD BeadChip, respectively. We identified different sets of candidate genes associated with litter size in different breeds: *BMPR1B, FBN1*, and *MMP2* in Wadi; *GRIA2, SMAD1*, and *CTNNB1* in Hu; *NCOA1* in Icelandic; *INHBB, NF1, FLT1, PTGS2*, and *PLCB3* in Finnsheep; *ESR2* in Romanov and *ESR1, GHR, ETS1, MMP15, FLI1*, and *SPP1* in Texel. Further annotation of genes and bioinformatics analyses revealed that different biological pathways could be involved in the variation in litter size of females: hormone secretion (FSH and LH) in Wadi and Hu, placenta and embryonic lethality in Icelandic, folliculogenesis and LH signaling in Finnsheep, ovulation and preovulatory follicle maturation in Romanov, and estrogen and follicular growth in Texel. Taken together, our results provide new insights into the genetic mechanisms underlying the prolificacy trait in sheep and other mammals, suggesting targets for selection where the aim is to increase prolificacy in breeding projects.

## Introduction

Reproduction is one of the most important traits in livestock production particularly for females. Selection for higher prolificacy in domestic sheep (*Ovis aries*) has led to variable litter size (LS) within and among breeds. For example, individual litter size of 1 to 8 has been recorded in the Hu sheep and Finnsheep (Yue, 1996; Davis et al., 2006a).

Previous studies reported that the exceptional prolificacy of the Booroola Merino was attributed to a single major gene, while a number of mutations of a major effect on litter size have been identified in other sheep breeds (**Table 1**; see also Xu and Li, 2017). Vage et al. (2013) detected a mutation FecG^F^ in gene *GDF9* strongly associated with litter size in Norwegian White Sheep and Finnish Landrace (Finnsheep) using a genome-wide association analysis. Demars et al. (2013) reported the mutations FecX^Gr^ in Grivette sheep and FecX^O^ in Olkuska sheep associated with the highly prolific phenotype by a genome-wide association analysis. Cao et al. (2016) found that nine candidate genes including the well-known FecB mutation played important roles in the variable litter size in Hu and Small-tailed Han sheep through methylated DNA-immunoprecipitation sequencing data. Miao et al. (2016) identified a set of differentially expressed genes (e.g., FecB) between low- and high-prolificacy breeds (Dorset vs. Small-tailed Han sheep) through implementing integrated analysis of miRNAs and lncRNAs. Lassoued et al. (2017) found the mutation FecX^Bar^ associated with the prolificacy in Tunisian Barbarine. Despite its great importance the genetic mechanisms of the high prolificacy trait in domestic sheep are still poorly understood, partly due to shortage of studies conducted across multiple prolific sheep breeds. To date, numerous fecundity-associated mutations have been identified in different sheep breeds, but very few mutations have been consistently detected across the breeds. Despite the reproduction of ewes can be affected by the complex interactions of environmental conditions (i.e., climate, density, and food abundance) (Wilson et al., 2009), previous studies suggested that genetic factor could play important roles in the variable litter size of ewes.

**Table 1:** Genetics variants associated with the fecundity in sheep.

Gene	Mutation	Name, allele symbol	Founder breeds	Reference
*BMP15*	V299D	Inverdale, FecXI	Romney, Inverdale	Galloway et al., 2000
	Q291Ter	Hanna, FecXH	Romney	Galloway et al., 2000
	S367I	Belclare, FecXB	Belclare	Hanrahan et al., 2004
	Q239R	Galway, FecXG	Belclare, Cambridge, Small-tailed Han	Hanrahan et al., 2004
	C321Y	Lacaune, FecXL	Lacaune	Bodin et al., 2007
	ΔP154S159	Rasa Aragonesa, FecXR	Rasa Aragonesa	Martinez-Royo et al., 2008; Monteagudo et al., 2009
	T317I	Grivette, FecXGr	Grivette (France)	Demars et al., 2013
	N337H	Olkuska, FecXO	Olkuska (Poland)	Demars et al., 2013
	c.301G > T, c.310insC, c.302_304delCTA	Barbarine, FecXBar	Tunisian Barbarine	Lassoued et al., 2017
	Unknown	Woodlands, FecXW	Woodlands	Feary et al., 2007
*BMPR1B*	Q249R	Booroola, FecBB	Booroola Merino, Garole, Javanese, Small-tailed Han, Wadi, Hu	Mulsant et al., 2001; Souza et al., 2001; Wilson et al., 2001; Chu et al., 2011; Zhang et al., 2011; Cao et al., 2016
*GDF9*	S395F	High Fertility, FecGH	Belclare, Cambridge	Hanrahan et al., 2004
	S427R	Thoka, FecGT	Icelandic	Nicol et al., 2009
	F345C	Embrapa, FecGE	Santa Ines	Silva et al., 2011
	V371M	FecGF	Norwegian White Sheep, Finnsheep Landrace, Belclare	Vage et al., 2013; Mullen and Hanrahan, 2014
	R315C	Vacaria, FecGV	Brazilian sheep	de Souza et al., 2012
	R87H	FecGI	Baluchi	Moradband et al., 2011
*B4GALNT2*		Lacaune, FecLL	Lacaune	Drouilhet et al., 2013
*Woodlands*		Wood-land, FecX2W	Coopworth	Davis, 2005
*OLKUSKA*			Olkuska	Davis, 2004
*BELLE-ILE*			Belle-Ile	Davis, 2005
*Unknown*		FecW		Davis et al., 2006b

**FIGURE 1:**
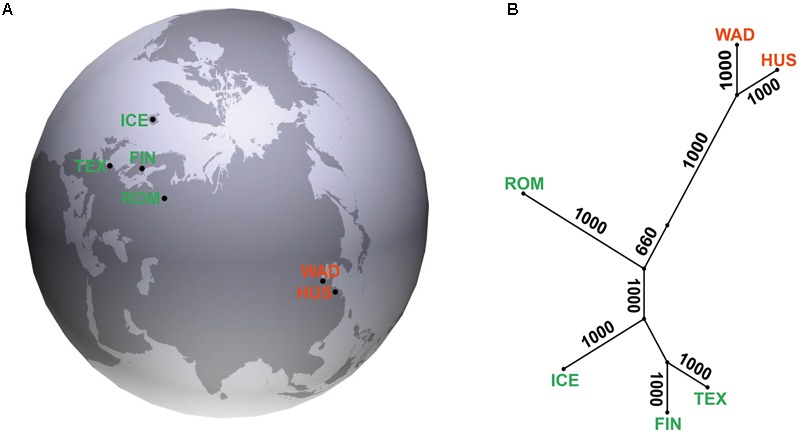
**(A)** Geographic locations for five sheep breeds of high (WAD, Wadi sheep; HUS, Hu sheep; ICE, Icelandic sheep; FIN, Finnsheep; and ROM, Romanov sheep) and one low (TEX, Texel sheep) prolificacy. **(B)** Neighbor-joining tree of the six sheep breeds with 1000 bootstrap replicates.

In this study, we conducted multiple independent genome-wide association studies (GWAS) on litter size in the sheep breeds of high (Wadi, Hu, Icelandic, Finnsheep, and Romanov) and low (Texel) prolificacy with a litter size ranging from 1 to 6 from different geographic regions (**Figure 1A**) and genetic origins (**Figure 1B**) of the world, respectively. Wadi sheep is a high-prolificacy native breed from the Shandong Province of China (Peng et al., 2017). Hu sheep is famous for early sexual maturity and high fecundity, and are distributed in the Taihu Lake area of Eastern China (Yue, 1996). Icelandic and Finnsheep (Finnish Landrace) sheep are northern European high-fecundity breeds (Mullen and Hanrahan, 2014; Eiriksson and Sigurdsson, 2017). Romanov sheep from the Volga Valley shows outstanding reproduction qualities: early sexual maturity, out-of-season breeding and extraordinary prolificacy (Deniskova et al., 2017). The Texel sheep is a relatively low-prolificacy breed originally from the island of Texel in the Netherlands and excels in muscle growth and lean carcasses (Casas et al., 2004). Our results will be important for further genetic improvement of the trait and for better understanding the molecular basis of reproduction in sheep as well as other mammals.

## Materials and Methods

### Sample Collection and Phenotyping

**FIGURE 2:**
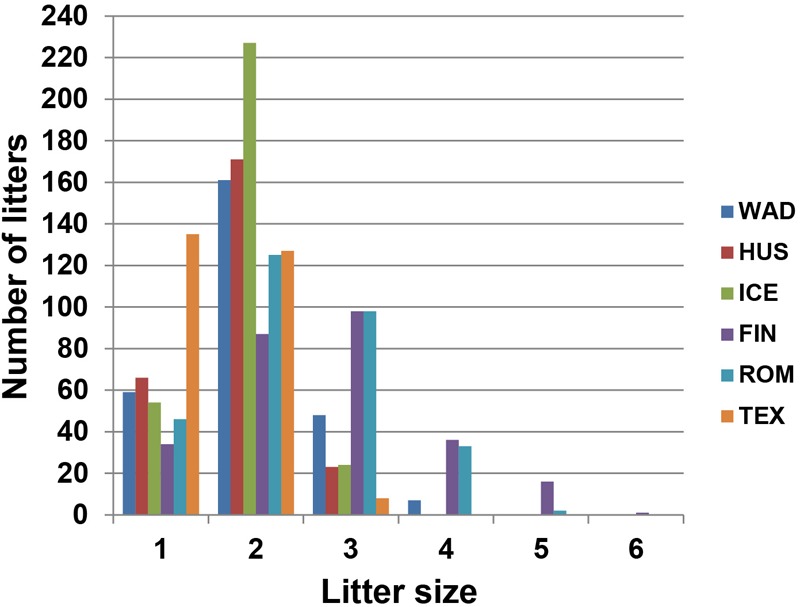
Phenotypic distribution of litter size in the six sheep breeds (WAD, Wadi sheep; HUS, Hu sheep; ICE, Icelandic sheep; FIN, Finnsheep; ROM, Romanov sheep; and TEX, Texel sheep).

A total of 522 ewes from five sheep breeds of high (Wadi, *n* = 160; Hu, *n* = 117; Icelandic, *n* = 54; Finnsheep, *n* = 54; and Romanov, *n* = 78) and one low (Texel, *n* = 59) prolificacy were collected from farms in China, Iceland, Finland, and Russia (**Figure 1A**). Animals included were as unrelated as possible based on analysis of pedigree records and farmers’ knowledge. Data for the phenotype of litter size and the total number of litters collected from farm records are shown in **Figure 2**. The litter size ranged from 1 to 6 based on parity from 1 to 11 in six sheep breeds. Genomic DNA was extracted from the ear marginal tissues following a standard phenol/chloroform method and was diluted to 50 ng/μl for the SNP BeadChip genotyping (Köchl et al., 2005), except for the Icelandic samples which were isolated from whole-blood using MasterPure^TM^ Complete DNA Purification Kit (Epicentre Biotech) following the manufacturers protocol.

### Genotyping and Quality Control

All the samples were genotyped using the Ovine Infinium HD BeadChip according to the manufacturer’s protocol. Genotypes of a total of 606,006 SNPs were obtained (genotype and phenotype datasets^1^). We implemented quality control of these SNPs using PLINK v1.07 software (Purcell et al., 2007). The SNPs or individuals were excluded if they met any of the criteria: (1) no chromosomal or physical location, (2) call rate < 0.95, (3) missing genotype frequency > 0.05, and/or (4) minor allele frequency (MAF) < 0.05. SNPs were excluded from the analysis if a *p*-value of Fisher’s exact test for Hardy–Weinberg equilibrium less than 0.001.

### Genetic Relationships and Population Structure

To investigate the genetic relationships and population structure among the six domestic sheep, we performed global *F*_ST_, neighbor-joining (NJ) tree and principle component analysis (PCA). The global *F*_ST_ value was calculated using GENEPOP v4.2 (Raymond and Rousset, 1995). The genetic distances between populations were calculated using an identity by state (IBS) similarity matrix (Kang et al., 2010). Then, the distances were used to construct a NJ tree with 1000 bootstraps using the package PHYLIP v.3.695 (Felsenstein, 1989). In addition, PCA was conducted using the SmartPCA program from the EIGENSOFT package version 4.2 (Patterson et al., 2006) based on the genotypes data.

### Genome-Wide Association Analysis

To explore genetic structure within the breeds, multidimensional scaling (MDS) analysis was performed based on the independent SNPs using PLINK v1.07. Firstly, we implemented the option of ‘indep-pairwise 50 5 0.05’ in PLINK v1.07, which calculated pairwise linkage disequilibrium (LD) in a 50-SNP-window shifted at a pace of five SNPs. If the LD estimate was *r*^2^ > 0.05, one of the pairs of SNPs was removed (Purcell et al., 2007). The independent SNPs retained by the LD criteria were then used in the MDS analysis, and the results were plotted using the GenABEL package in R v3.2.2 (Aulchenko et al., 2007).

We performed genome-wide association studies within five sheep breeds of high prolificacy (Wadi, Hu, Icelandic, Finnsheep, and Romanov) and one low prolificacy (Texel) using the case/control design. We ranked all individuals within the breeds according to their litter size from the highest to lowest. Then, we selected individuals from two tails for each breed as ‘case’ and ‘control,’ respectively. Based on the distribution of phenotypes, 114 samples (LS ≥ 2) in Wadi, 66 samples (LS ≥ 2) in Hu, 20 samples (LS > 2) in Icelandic, 37 samples (LS ≥ 2.5) in Finnsheep, 40 samples (LS ≥ 2.5) in Romanov and 28 samples (LS ≥ 1.6) in Texel sheep were selected as ‘cases,’ while 28 samples (LS = 1) in Wadi, 15 samples (LS = 1) in Hu, 15 samples (LS ≤ 1.75) in Icelandic, 9 samples (LS ≤ 2) in Finnsheep, 26 samples (LS ≤ 2) in Romanov and 14 samples (LS ≤ 1.33) in Texel sheep were selected as ‘controls.’ In the GWAS, we used the function of “qtscore” in the GenABEL package. Associated SNPs were identified at both the genome-wide and chromosome-wise significance levels (*p* < 0.05) after the Bonferroni correction (Bonferroni, 1936). To account for systematic biases caused by within-population substructure, the first and second dimensions from the MDS analyses were used as the covariates (Price et al., 2006). The correlation analysis between litter size and parity within breeds showed that there were significant effects between litter size and parity in four breeds (Wadi, Hu, Icelandic, and Texel), and the effect of parity 1 on litter size was less than that of parities 2 through 10 (**Supplementary Table S1** and **Supplementary Figure S1**). However, the parity of individuals within breeds was different, and we mainly focused on the mean of litter size of individual (total litter size/parity) in per breed. Therefore, we excluded the effect of parity from the model. The Quantile–Quantile (Q–Q) plots were visualized by plotting the distribution of obtained vs. expected genome-wide *p*-values. For genotype effect of potential SNPs on litter size in each breed, differences between means were analyzed by the Student’s *t*-test. The *p* < 0.05 was considered statistically significant. All the results were presented as mean ± standard error (SE). We implemented pairwise tests of linkage disequilibrium (LD) between the most significant SNPs and their flanking SNPs within approximately 1 Mb upstream and downstream using PLINK v1.07. Regional association plots were generated using the R package v3.2.2.

### Bioinformatics Analysis

We annotated the genes associated with litter size in each breed using the *O. aries* assembly Oar_v.4.0^2^. Further, we submitted the genes to the DAVID (database for annotation, visualization and integrated discovery) database^3^ for gene ontology (GO) enrichment and pathways analyses (Huang et al., 2009a,b). The *p*-value of 0.1 and at least two genes from the input gene list in the enriched category were considered for the enriched GO terms. Also, we investigated the protein–protein interaction network for the candidate genes using the STRING database version 10.5 (Szklarczyk et al., 2017). In addition, differential expressions of the candidate genes in various tissues were examined using the EMBL-EBI Expression Atlas database^4^ (Petryszak et al., 2016).

## Results

### Population Relationship and Differentiation

Pairwise *F*_ST_ value varied from 0.023 to 0.104 among the populations with the least genetic differentiation observed between Wadi and Hu sheep breeds (**Supplementary Table S2**). The NJ tree showed that these breeds were clustered into two major groups according to their Chinese and European origins (**Figure 1B**). A similar geographic pattern was seen in the PCA analyses with the grouping of Wadi and Hu sheep separated from the other four European breeds (**Supplementary Figure S2**).

### Genome-Wide Association Analysis

After the quality control, 508,444 SNPs and 114 individuals (91 cases vs. 23 controls) in Wadi, 506,031 SNPs and 80 individuals (66 cases vs. 14 controls) in Hu, 443,125 SNPs and 23 individuals (8 cases vs. 15 controls) in Icelandic, 492,165 SNPs and 37 individuals (28 cases vs. 9 controls) in Finnsheep, 465,794 SNPs and 38 individuals (29 cases vs. 9 controls) in Romanov, 475,955 SNPs and 39 individuals (28 cases vs. 11 controls) in Texel sheep were retained in the working dataset for the GWAS. We did find several animals outlying the clusters of cases, which might cause biases in the association analyses (**Supplementary Figure S3**). We have repeated the association analyses without these animals, and found the results are very similar. Thus, we did not exclude these animals in the association analyses due to the small sample size for the breeds. The resulting genomic inflation factors were equal to 1.07 in Wadi, 1.14 in Hu, 1.12 in Icelandic, 1.14 in Finnsheep, 1.10 in Romanov, and 1.05 in Texel sheep, suggesting well-controlled population stratifications (**Supplementary Figure S4**).

**FIGURE 3:**
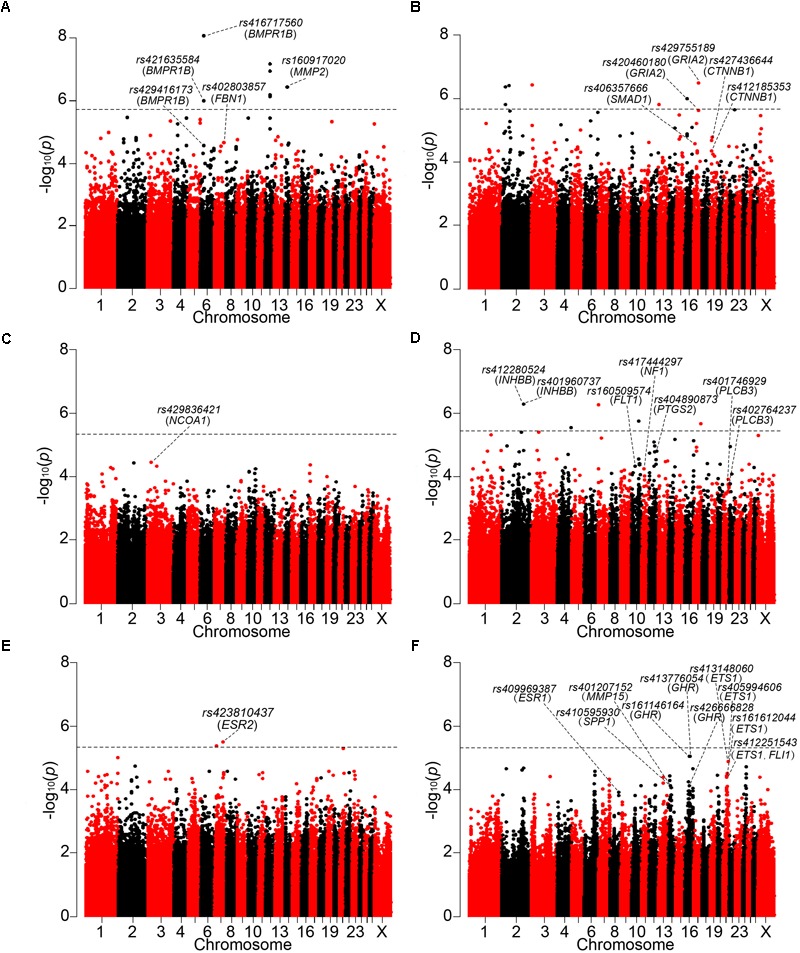
Manhattan plots of GWAS are shown on **(A)** Wadi, **(B)** Hu, **(C)** Icelandic, **(D)** Finnsheep, **(E)** Romanov and **(F)** Texel sheep. The 5% genome-wide significant threshold value is indicated by a dotted line. The significant SNPs surrounding the genes previously reported to be associated with reproduction are annotated at the chromosome-wise and genome-wide 5% significance after the Bonferroni correction.

**FIGURE 4:**
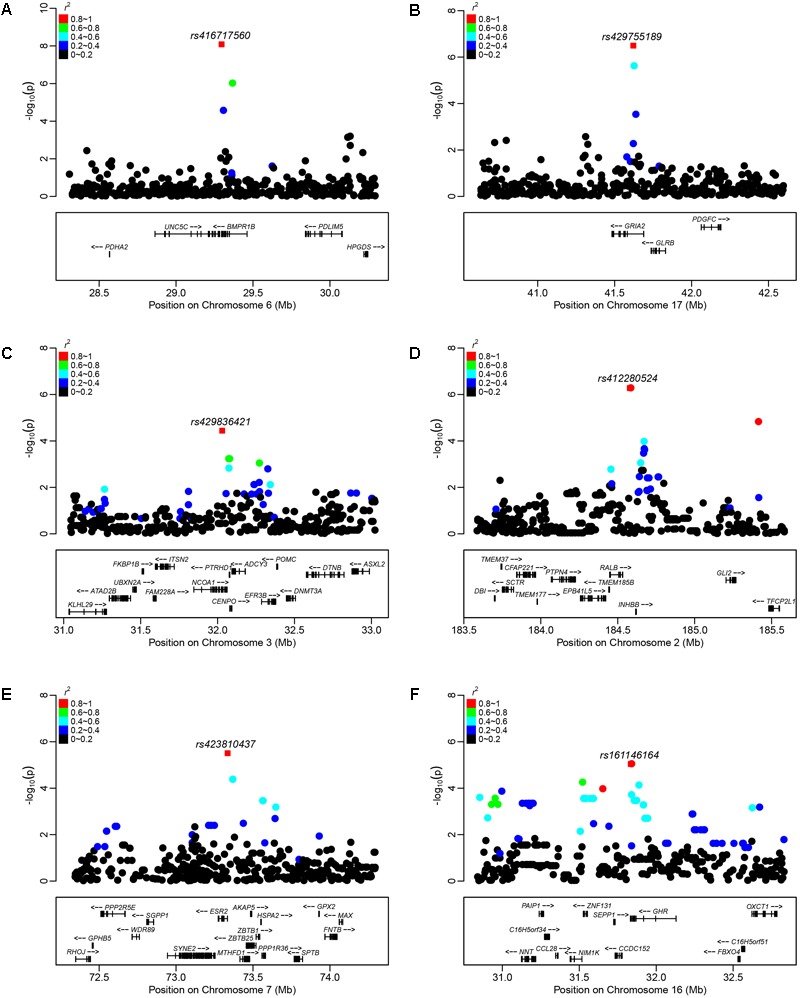
Plots of regional association results for the top significant SNP (red square) and their near SNPs in **(A)** Wadi, **(B)** Hu, **(C)** Icelandic, **(D)** Finnsheep, **(E)** Romanov, and **(F)** Texel sheep. Different colors represent the *r^2^* values of pair-wise LD estimates.

**FIGURE 5:**
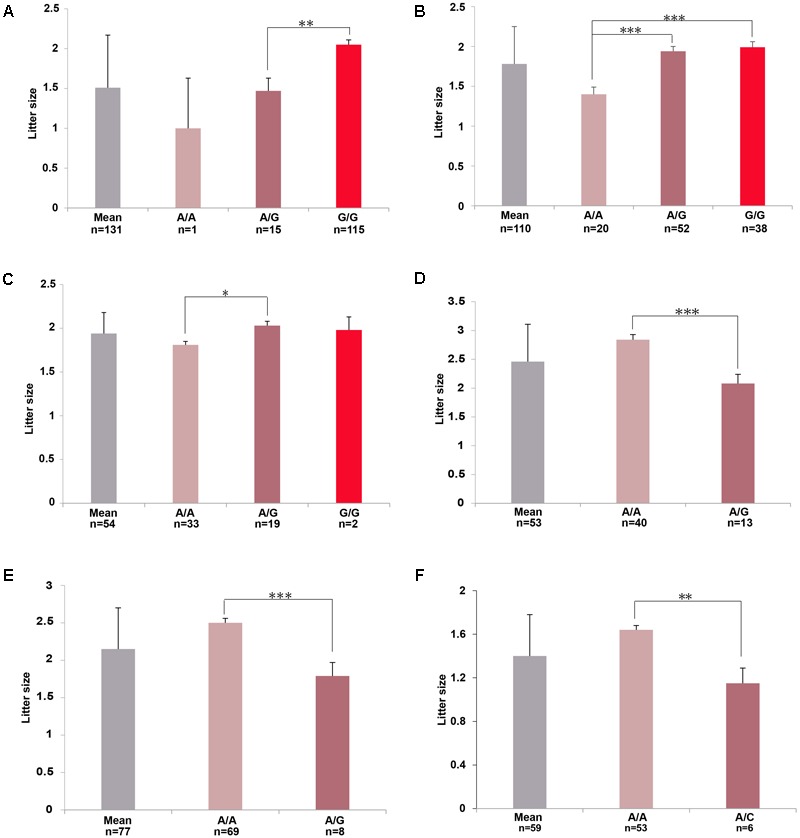
Genotypic distributions of the top significant SNPs for the litter size (LS) phenotype in **(A)** Wadi, **(B)** Hu, **(C)** Icelandic, **(D)** Finnsheep, **(E)** Romanov, and **(F)** Texel sheep, respectively. The means LS were calculated for various breeds. Number of ewes per group of genotype is mentioned. Pairwise statistical comparisons between means of genotype’s clades were performed using Student’s *t*-test. ^∗^*p* < 0.05, ^∗∗^*p* < 0.01, ^∗∗∗^*p* < 0.001.

In Wadi sheep, we detected 59 and 8 SNPs at the chromosome-wise and genome-wide (*p* < 1.92 × 10^-6^) 5% significance after the Bonferroni correction, respectively (**Figure 3A** and **Supplementary Tables S3, S4**). We observed a high level of LD between the top significant SNP *rs416717560* and *rs421635584* located in gene *BMPR1B* (**Figure 4A**). For the SNP *rs416717560*, average litter size of individuals with the G/G genotype (*n* = 115, LS = 2.05 ± 0.06) was significantly (*p* < 0.01) higher than that of the ewes with the A/G (*n* = 15, LS = 1.47 ± 0.16) genotype (**Figure 5A**). Also, we found three additional significant SNPs (*rs429416173, rs402803857*, and *rs160917020*) neighboring genes *BMPR1B, FBN1*, and *MMP2* (**Table 2** and **Supplementary Table S3**).

**Table 2:** Genome-wide and chromosome-wise significant SNPs and associated genes.

Population	SNP	Chr	Position (bp)	MAF	*p*-unadjusted	*p*-adjusted	Genes	Location
Wadi	*rs416717560^∗^*	6	29295803	0.07	3.65E-08	8.19E-09	*BMPR1B*^1^	3′UTR
	*rs421635584^∗^*	6	29361782	0.05	4.36E-06	9.78E-07	*BMPR1B*^1^	Intron
	*rs429416173*	6	29302788	0.2	7.55E-05	2.75E-05	*BMPR1B*^1^	CDS
	*rs402803857*	7	58598895	0.1	4.96E-05	2.93E-05	*FBN1*^1^	Intron
	*rs160917020^∗^*	14	23133427	0.19	1.10E-06	3.71E-07	*MMP2*	Downstream
Hu	*rs429755189^∗^*	17	41621298	0.43	1.94E-06	3.21E-07	*GRIA2*^1^	Intron
	*rs420460180*	17	41621269	0.29	8.50E-06	2.43E-06	*GRIA2*^1^	Intron
	*rs406357666*	17	12487861	0.19	1.40E-05	2.66E-05	*SMAD1*^1^	Intron
	*rs427436644*	19	13639996	0.32	7.69E-05	2.14E-05	*CTNNB1*	Downstream
	*rs412185353*	19	13641870	0.33	1.51E-04	4.49E-05	*CTNNB1*	Downstream
Icelandic	*rs429836421*	3	32030054	0.16	4.55E-05	3.63E-05	*NCOA1*^1^	Intron
Finnsheep	*rs412280524^∗^*	2	184578329	0.09	2.62E-05	5.32E-07	*INHBB*	Downstream
	*rs401960737^∗^*	2	184579671	0.09	2.62E-05	5.32E-07	*INHBB*	Downstream
	*rs160509574*	10	31933001	0.27	1.50E-05	4.71E-05	*FLT1*^1^	Intron
	*rs417444297*	11	18552961	0.11	4.20E-05	5.65E-05	*NF1*	Downstream
	*rs404890873*	12	65662842	0.05	1.87E-04	1.59E-05	*PTGS2*	Upstream
	*rs401746929*	21	41915064	0.08	1.85E-03	1.75E-04	*PLCB3*	Upstream
	*rs402764237*	21	41919836	0.08	1.85E-03	1.75E-04	*PLCB3*	Upstream
Romanov	*rs423810437^∗^*	7	73335157	0.07	1.65E-05	3.12E-06	*ESR2*^1^	5′ flanking region
Texel	*rs409969387*	8	75353388	0.08	1.11E-03	1.21E-04	*ESR1*	Intron
	*rs410595930*	14	23645021	0.06	1.33E-04	1.46E-04	*SPP1*^1^	Intron
	*rs401207152*	14	25147418	0.06	1.33E-04	1.46E-04	*MMP15*	Downstream
	*rs161146164*	16	31834495	0.06	1.33E-04	9.11E-06	*GHR*^1^	CDS
	*rs413776054*	16	31834942	0.06	1.33E-04	9.11E-06	*GHR*	CDS
	*rs426666828*	16	31882869	0.18	1.88E-04	7.54E-05	*GHR*^1^	Intron
	*rs413148060*	21	30950537	0.15	1.02E-04	4.17E-05	*ETS1*	Upstream
	*rs405994606*	21	31001548	0.15	1.02E-04	4.17E-05	*ETS1*^1^	Intron
	*rs161612044*	21	31009743	0.14	5.41E-04	1.01E-04	*ETS1*^1^	Intron
	*rs412251543*	21	31178275	0.1	4.01E-03	1.46E-04	*ETS1*/*FLI1*	Upstream/Downstream

*For genes the best SNP of which is located outside of upstream/downstream 150 kb region. Chr., chromosome; MAF, Minor Allele Frequency. The p-unadjusted corresponds to exact p for the Fisher’s test. The p-adjusted corresponds to the corrected significance of GWAS after principle component adjustment. The SNPs with symbol (^∗^) denote that bonferroni-corrected genome-wide significant SNPs. The genes with symbol (^*1*^) denote that the SNPs are intragenic, otherwise they are the nearest genes upstream and downstream of the tested SNPs.*

In Hu sheep, we identified 98 and 9 SNPs at the chromosome-wise and genome-wide (*p* < 2.18 × 10^-6^) 5% significance after Bonferroni correction (**Figure 3B** and **Supplementary Tables S3, S4**). The regional plot showed that the top significant SNPs *rs429755189* and *rs420460180* on chromosome 17 were in an LD block that contained gene *GRIA2* (**Figure 4B**). For the *rs429755189*, average litter size of individuals with the genotypes G/G (*n* = 38, LS = 1.99 ± 0.07) and A/G (*n* = 52, LS = 1.94 ± 0.06) were significantly (*p* < 0.001) higher than that of ewes with the genotype A/A (*n* = 20, LS = 1.40 ± 0.09) in the present population (**Figure 5B**). Among these significant SNPs, 3 (*rs406357666, rs427436644* and *rs412185353*) are located within the genes *SMAD1* and *CTNNB1* (**Table 2** and **Supplementary Table S3**).

In Icelandic sheep, we found 22 SNPs at the chromosome-wise 5% significance after the Bonferroni correction (**Figure 3C** and **Supplementary Tables S3, S4**). The top significant SNP *rs429836421* on chromosome 3 was located within gene *NCOA1* (**Figure 4C**). For *rs429836421*, average litter size of individuals with the A/G genotype (*n* = 19, LS = 2.03 ± 0.05) is significantly (*p* < 0.05) higher than that of the ewes with the genotype A/A (*n* = 33, LS = 1.81 ± 0.04) (**Figure 5C**).

In Finnsheep, we detected 102 and 6 SNPs at the chromosome-wise and genome-wide (*p* < 3.64 × 10^-6^) 5% significance after the Bonferroni correction, respectively (**Figure 3D** and **Supplementary Tables S3, S4**). The regional plot revealed strong LD between the top significant SNP *rs412280524* and its neighboring SNPs *rs401960737* and *rs407751830* harbored gene *INHBB* (**Figure 4D**). For the SNP *rs412280524*, litter size of ewes with the genotype A/A (*n* = 40, LS = 2.84 ± 0.09) is significantly (*p* < 0.001) higher than that of the ewes with the genotype A/G (*n* = 13, LS = 2.08 ± 0.16) (**Figure 5D**). Also, five additional significant SNPs (*rs160509574, rs417444297, rs404890873, rs401746929*, and *rs402764237*) were found to be located near to genes *FLT1, NF1, PTGS2*, and *PLCB3* (**Table 2** and **Supplementary Table S3**).

In Romanov sheep, we identified 77 and 2 SNPs at the chromosome-wise and genome-wide (*p* < 4.56 × 10^-6^) 5% significance after the Bonferroni correction (**Figure 3E** and **Supplementary Tables S3, S4**). The top significant SNP *rs423810437* on chromosome 7 was in the gene *ESR2* (**Figure 4E**). For *rs423810437*, litter size of ewes with the genotype A/A (*n* = 69, LS = 2.50 ± 0.06) is significantly (*p* < 0.001) higher than that of the ewes with the genotype A/G (*n* = 8, LS = 1.79 ± 0.18) (**Figure 5E**).

In Texel sheep, we observed 133 SNPs at the chromosome-wise 5% significance after the Bonferroni correction (**Figure 3F** and **Supplementary Tables S3, S4**). The regional plot showed that the top significant SNPs *rs161146164* and *rs413776054* on chromosome 16 were in a strong LD region containing one functional gene *GHR* (**Figure 4F**). For *rs161146164*, litter size of ewes with the genotype A/A (*n* = 53, LS = 1.64 ± 0.05) is significantly (*p* < 0.01) higher than that of the ewes with the genotype A/C (*n* = 6, LS = 1.15 ± 0.14) (**Figure 5F**). The two mutations (rs161146164, Asn > His; rs413776054, Pro > Ser) cause the amino acid change in coding region of the GHR gene. In addition, we found eight additional significant SNPs (*rs426666828, rs409969387, rs410595930, rs401207152, rs413148060, rs405994606, rs161612044*, and *rs412251543*) surrounding genes *ESR1, ETS1, FLI1, SPP1*, and *MMP15* (**Table 2** and **Supplementary Table S3**).

In addition to the source breed where the target SNPs have been detected, we further assessed genotype effect of the most significant SNPs on litter size in the other five sheep breeds. In general, genotypes of the target SNPs did not show significant association with increased litter size in the breeds other than the source breed (**Supplementary Table S7**). Nevertheless, we observed some exceptions. For example, the genotype A/G of *rs429836421*, which was identified in Icelandic sheep, showed significant associations with increased litter size in both Icelandic and Hu sheep breeds. However, a lack of homozygotes for the SNPs such as the genotype G/G for *rs412280524* in Finnsheep, G/G for *rs423810437* in Romanov and C/C for *rs161146164* in Texel sheep could be because of low frequency of the mutations and small sample size.

### Bioinformatics Analysis

**FIGURE 6:**
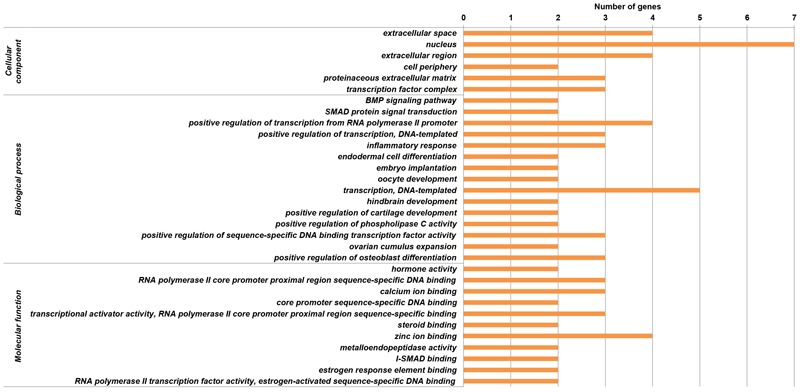
Gene ontology (GO) enrichments based on the functional genes surrounding the significant SNPs at the chromosome-wise 5% level.

**FIGURE 7:**
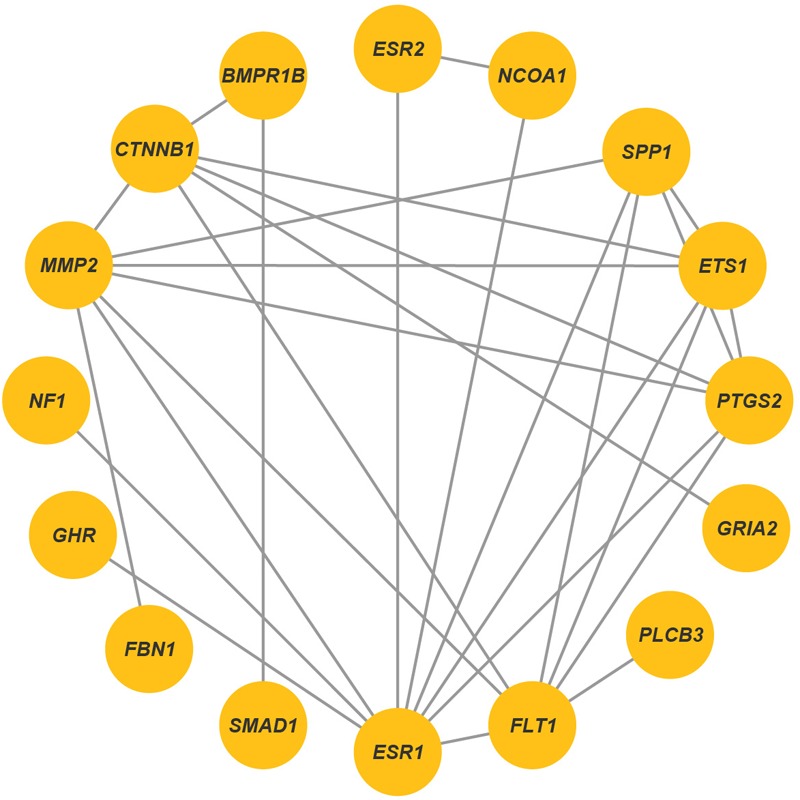
Protein–protein interaction networks identified by using STRING database. Each line indicated known signaling pathways and protein complexes.

**FIGURE 8:**
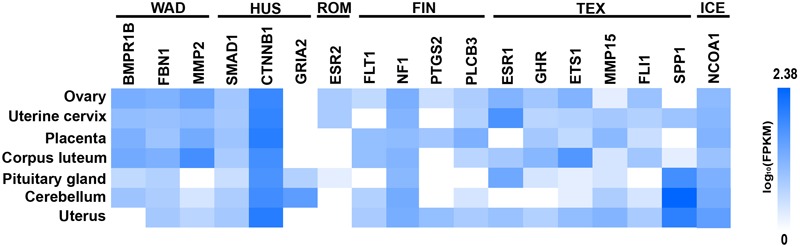
Heatmap of the candidate genes identified from six sheep breeds (WAD, Wadi sheep; HUS, Hu sheep; ICE, Icelandic sheep, FIN, Finnsheep, ROM, Romanov sheep, and TEX, Texel sheep) enriched for expression in different ewes tissues deposited in the EBI Gene Expression Atlas database. The FPKM (fragments per kilobase of transcript per million mapped reads) value is used to measure the expression level.

We found significantly (*p* < 0.1) enriched GO terms associated with reproduction for the candidate genes. The GO clusters were primarily enriched in the categories of ovarian and oocyte development (*PTGS2, BMPR1B, INHBB, CTNNB1, MMP2, MMP15, FBN1, GHR*, and *SPP1*), phospholipase C activity (*FLT1* and *ESR1*), SMAD protein (*INHBB* and *SMAD1*) and BMP signaling (*SMAD1* and *BMPR1B*) and positive regulation of transcription (*NCOA1, FLI1, ESR1, ESR2, CTNNB1, ETS1*, and *BMPR1B*), all of which are involved in the folliculogenesis, follicle growth and granulosa cell proliferation (**Figure 6** and **Supplementary Table S5**). Another relevant GO category was hindbrain development (*SMAD1* and *CTNNB1*), which participated in regulating ovulation (Baird et al., 2006). In addition, we detected 11 genes (i.e., *PLCB3, ESR1, ESR2, MMP2, NCOA1, CTNNB1, INHBB, SMAD1, BMPR1B, PTGS2*, and *GRIA2*) involved in estrogen, thyroid hormone, TGF-beta, retrograde endocannabinoid and hippo signaling pathways, and these pathways played important roles in regulating follicle growth and ovulation in livestock (**Supplementary Table S5**). However, we observed different GO terms for the candidate genes in different sheep breeds. For example, I-SMAD binding were enriched in Hu sheep, and chromatin binding were enriched in Texel sheep (**Supplementary Table S6**). In the gene network analysis, we observed that 16 genes (i.e., *BMPR1B, FBN1, MMP2, SMAD1, CTNNB1, GRIA2, NCOA1, FLT1, NF1, PTGS2, PLCB3, ESR2, ESR1, ETS1, SPP1*, and *GHR*) showed protein–protein interactions in the network (**Figure 7**). Expression data further showed that the genes *BMPR1B, FBN1, MMP2, GRIA2, SMAD1, CTNNB1, NCOA1, NF1, FLT1, PTGS2, PLCB3, ESR2, ESR1, GHR, ETS1, MMP15, FLI1*, and *SPP1* were either highly or moderately expressed in reproduction-related tissues such as ovary, uterine cervix, placenta, corpus luteum, cerebellum, pituitary gland or uterus in sheep (**Figure 8**). Also, gene *INHBB* showed a high expression in ovary and uterus of *Mus musculus*^5^.

## Discussion

In this study, we conducted multiple independent GWAS in different sheep breeds to investigate the genetic mechanisms underlying the litter size in sheep. Coupled with population relationship and bioinformatics analyses, the GWAS identified different genes associated with the litter size in different breeds and revealed their differentially genetic regulation mechanisms associated with follicle growth and ovulation in the reproduction of ewes.

**FIGURE 9:**
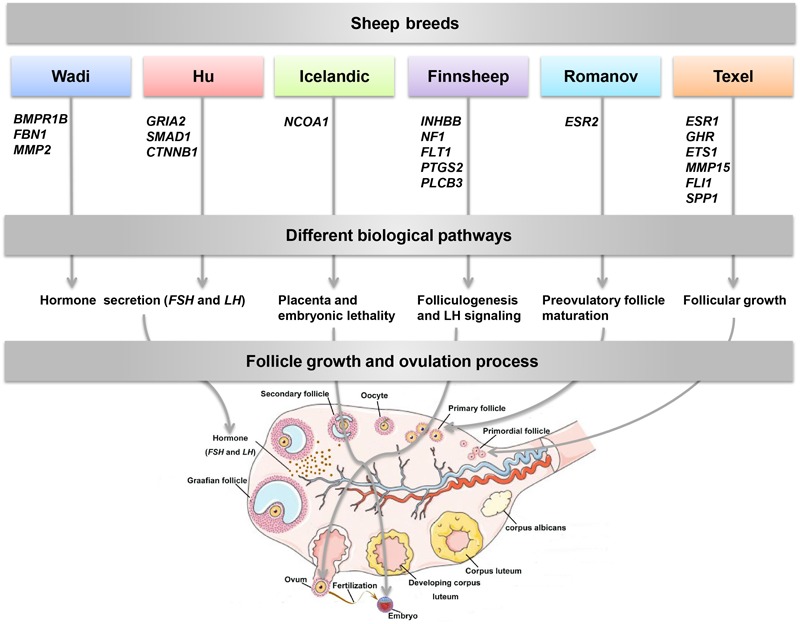
Follicle growth and ovulation process for the role of the candidate genes identified from six sheep breeds in litter size.

The diverse biological pathways identified from the novel genes annotation play an important role in follicle growth and ovulation of females in different sheep breeds (**Figure 9**). The three genes identified in Wadi sheep, *BMPR1B, FBN1*, and *MMP2*, all play a crucial role in regulating hormone secretion (Mulsant et al., 2001; Basini et al., 2011; Zhang et al., 2011; Zhai et al., 2013). For example, *BMPR1B* gene can lead to an increased density of the follicle-stimulating hormone (FSH) and luteinizing hormone (LH) receptors with a concurrent reduction in apoptosis to increase the ovulation rate of ewes (Regan et al., 2015; Hu et al., 2016). As the main component of microfibrils in the extracellular matrix, the gene *FBN1* regulates cumulus cell apoptosis by reducing the expression level of *BMP15* involved in estrogen signaling in porcine ovaries (Zhai et al., 2013). The *MMP2* gene plays a key role in ovulation and follicle atresia by regulating FSH and insulin like growth factor 1 (*IGF1*) (Knapp and Sun, 2017). In Hu sheep, the three genes *GRIA2, SMAD1*, and *CTNNB1* are related to estrogen response element (Chang et al., 2013; Kumar et al., 2016; Vastagh et al., 2016). For example, the gene *GRIA2* has been shown to participate in the glutamatergic pathway that regulates gonadotropin-releasing hormone (GnRH), a known prerequisite of the subsequent hormonal cascade inducing the ovulation in mice (Vastagh et al., 2016). The gene *SMAD1* encodes an intracellular BMP signaling molecule, which is involved in mediating ovulation rate of ewes (Xu et al., 2010). The *CTNNB1* gene enhances FSH and LH actions in follicles by stimulating WNT/CTNNB1 pathway and G protein-coupled gonadotropin receptors in female (Fan et al., 2010). In Icelandic sheep, the gene *NCOA1* can alter the expression of multiple key genes *PBP, AIB3*, and *FGFR2*, which are important for aberrant labyrinth morphogenesis of the placenta and embryonic lethality (Chen et al., 2010; Huang et al., 2011). In Finnsheep, the five candidate genes *INHBB, NF1, FLT1, PTGS2*, and *PLCB3* played important roles in the development of folliculogenesis and LH signaling (Ding et al., 2006; Tal et al., 2014; De Cesaro et al., 2015; Ben Sassi et al., 2016; Cadoret et al., 2017). For example, the *INHBB* gene encodes an inhibitor of apoptosis, which regulates porcine ovarian follicular atresia (Terenina et al., 2017). The coding region of gene *NF1* presents non-CpG methylation in the murine oocyte, which plays a critical role in mammalian development (Haines et al., 2001). The *FLT1* gene has an important role in the activity of vascular endothelial growth factor that linked to folliculogenesis (Celik-Ozenci et al., 2003). The *PTGS2* gene plays a critical role in the ovulation by stimulating LH signaling in zebrafish (Tang et al., 2017). The *PLCB3* gene is highly expressed in bovine cells of the ovulatory-sized follicles, with the role of activating LH/LHR signaling (Castilho et al., 2014). In Romanov sheep, the gene *ESR2* activates ovulation and regulates preovulatory follicle maturation through regulating estrogen response element (Laliotis et al., 2017; Rumi et al., 2017). In Texel sheep, the six candidate genes *ESR1, GHR, ETS1, MMP15, FLI1*, and *SPP1* are relevant to estrogen and follicular growth (Putnova et al., 2001; Bachelot et al., 2002; Munoz et al., 2007; Xiao et al., 2009; Hatzirodos et al., 2015; Ogiwara and Takahashi, 2017). As a key gene affecting estrogen biosynthesis, *ESR1* gene functions similarly to *ESR2*, and is critical for follicular growth and successful ovulation in ewes (Foroughinia et al., 2017). The *GHR* gene plays a role in follicular growth through stimulating *IGF1* in mice (Bachelot et al., 2002). The *ETS1* gene was linked to the regulator of protein signaling protein-2 (RGS2) involved in the ovulation in bovine (Sayasith et al., 2014). As a proteolytic enzyme gene, the *MMP15* gene has been shown to mediate LH and its receptor in the preovulatory follicles of teleost medaka (Ogiwara and Takahashi, 2017). The *FLI1* gene encodes a critical transcription factor, which regulates gene *ETS1* (Vo et al., 2017). The *SPP1* gene accounts for establishing and maintaining cellular interactions between steroidogenic and non-steroidogenic cells during the development of corpus luteum (Poole et al., 2013). In addition, the GO categories as well as protein–protein network and expression analysis showed that these genes played an essential role in follicle growth and ovulation of ewes. However, further expression analyses of these genes in each breed are necessary in future study. Taken together, the apparent difference for the litter size among the breeds might be explained by diverse regulation mechanisms.

Also, we calculated genetic differentiation among populations using the global *F*_ST_, PCA, and NJ tree methods to obtain a refined picture of population genetic relationships. The result showed that the genetic groups were consistent with the geographic origins of the breeds. The different genetic mechanisms associated with physiological processes for the litter size among sheep breeds could be related to the various environments in different geographic regions.

We noticed that previous studies had identified several genes of major effect such as *BMPR1B, BMP15*, and *GDF9* for the prolificacy in ewes (**Table 1**). Different from early investigations, we detected a set of novel genes for the litter size in ewes. The main reason could be that most of early studies are based on genome-wide selection tests between prolific and non-prolific breeds using a lower density of SNPs. Instead, here we implemented GWAS within specific sheep breeds of high or low prolificacy using a high density SNP BeadChip array, which should lead to more reliable associations. In addition, the difference in threshold value used to define the ‘case’ and ‘control’ groups for each breed was also another potentially influential factor. When we implemented the GWAS using a two-step approach via the general linear model and genome-wide efficient mixed-model analysis (GEMMA), we did not find interesting candidate genes associated with reproduction across the six breeds (see **Supplementary Material** for further details). The fact that no candidate genes associated with reproduction were detected could be due to that the power to detect such associations will be weak when treating the trait of interest as quantitative given the small sample size. Also, these populations could have been subjected to selection on litter size through environmental variables such as climate and diet. However, we did not obtain data for local environmental variables in our data. Thus, environmental variables as well as the age of reproduction for the ewes were not taken into account in the model of the GWAS, which would be essential for future study.

## Conclusion

We revealed a set of novel functional genes for the litter size in different sheep breeds across the world. Our results suggested differentially genetic regulation mechanisms for the functional genes in the reproduction of sheep. The significant SNPs and genes identified here are useful for future molecular-based breeding for a higher fertility. Also, our results provide important insights into the regulation of reproduction in sheep and other mammals.

## Author Contributions

M-HL conceived and designed the project. FW, Z-QS, Y-LR, MS, EE, JH, JK, and TK collected the samples. X-LX extracted the DNA. JK provided help in Beadchip genotyping. S-SX and LG analyzed the data. S-SX wrote the paper with contributions from M-HL. All authors reviewed and approved the final manuscript.

## Conflict of Interest Statement

The authors declare that the research was conducted in the absence of any commercial or financial relationships that could be construed as a potential conflict of interest.

## Footnotes

1 https://www.animalgenome.org/repository/pub/CAAS2018.0302/

2 http://www.ncbi.nlm.nih.gov/genome?term=ovis%20aries

3 https://david.ncifcrf.gov/

4 https://www.ebi.ac.uk/gxa/home/

5 https://www.ebi.ac.uk/gxa/home/

## Supplementary Material

FIGURE S1 Parity effect for litter size in the six breeds. X-axis is labeled as the number of parity and Y-axis represents litter size. Pairwise statistical comparisons between means of litter size in parity’s clades were performed using Student’s t-test. ^∗^*p* < 0.05; ^∗∗^*p* < 0.01, and ^∗∗∗^*p* < 0.001.

FIGURE S2 Principle component plots for 522 ewes from the six sheep breeds (WAD: Wadi sheep, HUS: Hu sheep, ICE: Icelandic sheep, FIN: Finnish sheep, ROM: Romanov sheep, and TEX: Texel sheep), respectively.

FIGURE S3 Multidimensional scaling (MDS) plots in **(a)** Wadi, **(b)** Hu, **(c)** Icelandic, **(d)** Finnish, **(e)** Romanov, and **(f)** Texel sheep. The red squares indicate animals from the case group (highly prolific ewes), and the purple dots represent animals in the control group (normally prolific ewes).

FIGURE S4 Q–Q (quantile–quantile) plots of GWAS in **(a)** Wadi, **(b)** Hu, **(c)** Icelandic, **(d)** Finnish, **(e)** Romanov, and **(f)** Texel sheep. Gray and black rings represent association statistics before and after correction for population stratification, respectively.

TABLE S1 Parity effect for litter size and pairwise statistical comparisons between means of litter size in parity’s clades in the six breeds.

TABLE S2 Pairwise *F*_ST_ value among six breeds.

TABLE S3 Bonferroni-corrected 5% chromosome-wise significance threshold in the six sheep breeds, respectively.

TABLE S4 Bonferroni-corrected genome-wide and chromosome-wise significant SNPs and their nearest gene based on the GWAS.

TABLE S5 GO enrichment analysis of the genes associated with the target SNPs at the chromosome-wise level as identified by the GWAS.

TABLE S6 GO enrichments of the novel genes identified by the GWAS at the chromosome-wise level for the six sheep breeds, respectively.

TABLE S7 Genotype effects of the most significant SNPs on litter size in six sheep breeds, respectively.
