# Supplementary figures and images for: Genome-Wide Association Analyses Highlight the Potential for Different Genetic Mechanisms for Litter Size Among Sheep Breeds
Source: Front Genet. 2018 Apr 10;9:118. doi: 10.3389/fgene.2018.00118 (PMC5902979; doi:10.3389/fgene.2018.00118)

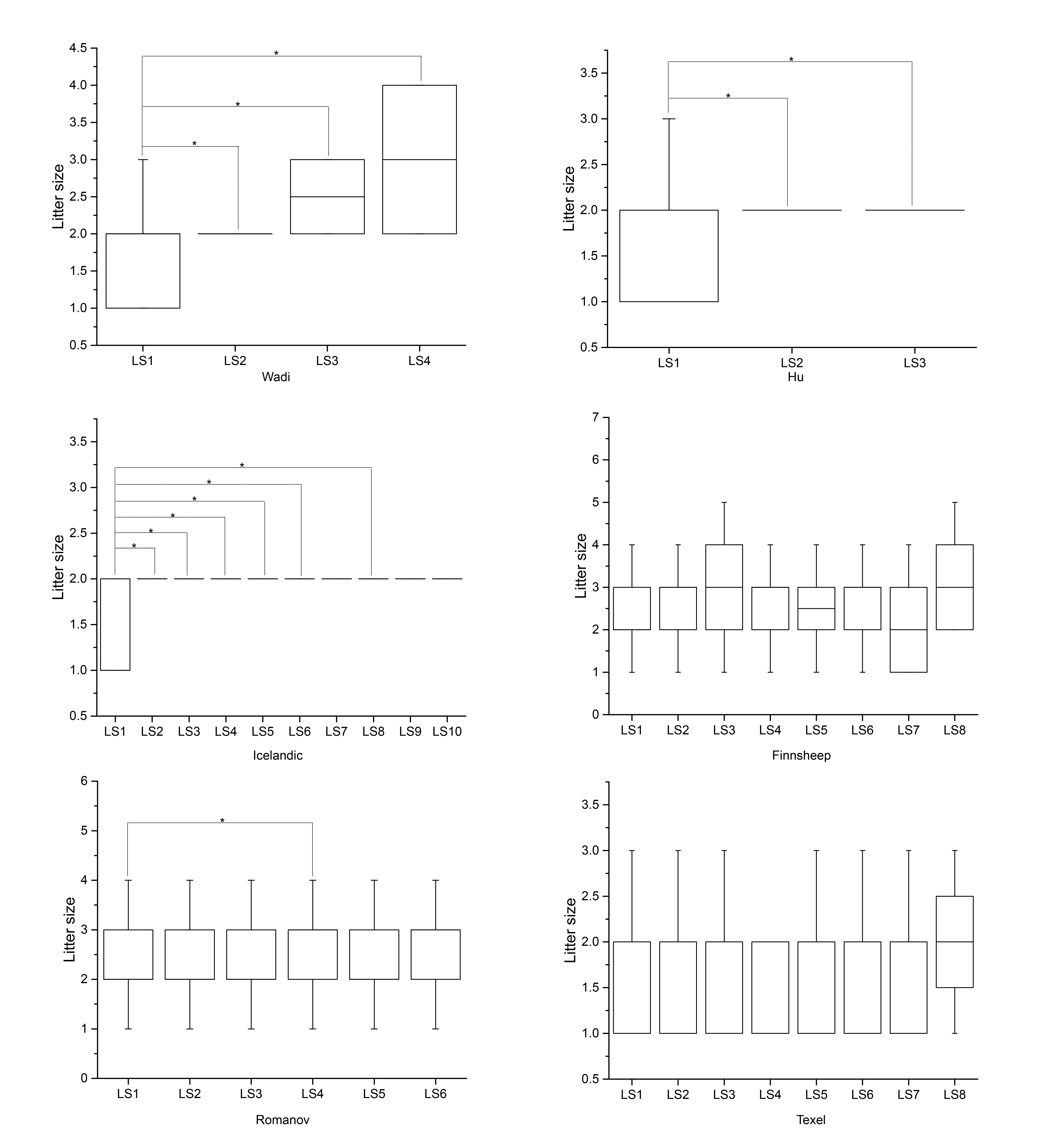

Supplement: FIGURE S1 — Parity effect for litter size in the six breeds. X-axis is labeled as the number of parity and Y-axis represents litter size. Pairwise statistical comparisons between means of litter size in parity’s clades were performed using Student’s t-test. ∗p < 0.05; ∗∗p < 0.01, and ∗∗∗p < 0.001. [file Image_1.JPEG]

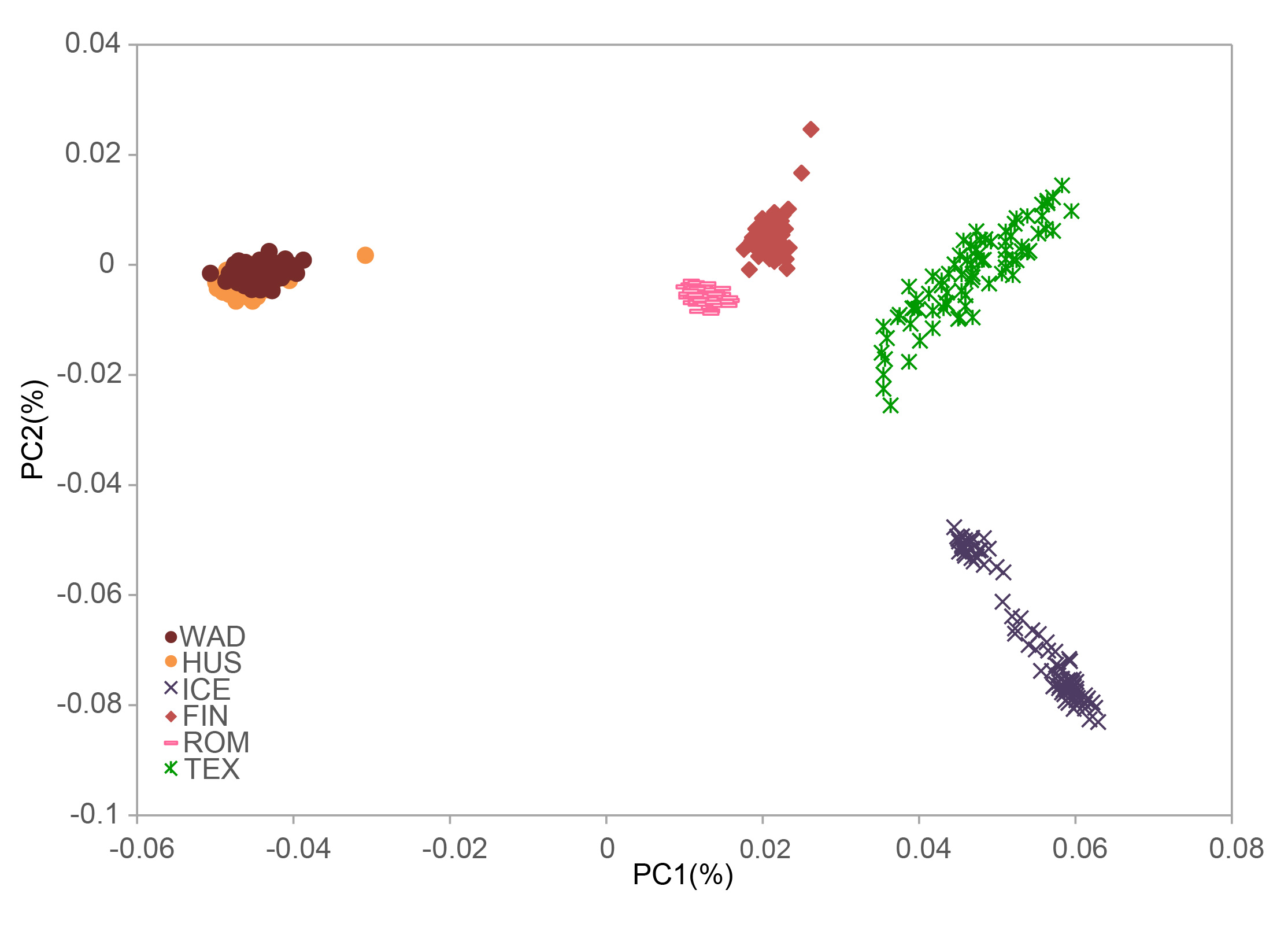

Supplement: FIGURE S2 — Principle component plots for 522 ewes from the six sheep breeds (WAD: Wadi sheep, HUS: Hu sheep, ICE: Icelandic sheep, FIN: Finnish sheep, ROM: Romanov sheep, and TEX: Texel sheep), respectively. [file Image_2.JPEG]

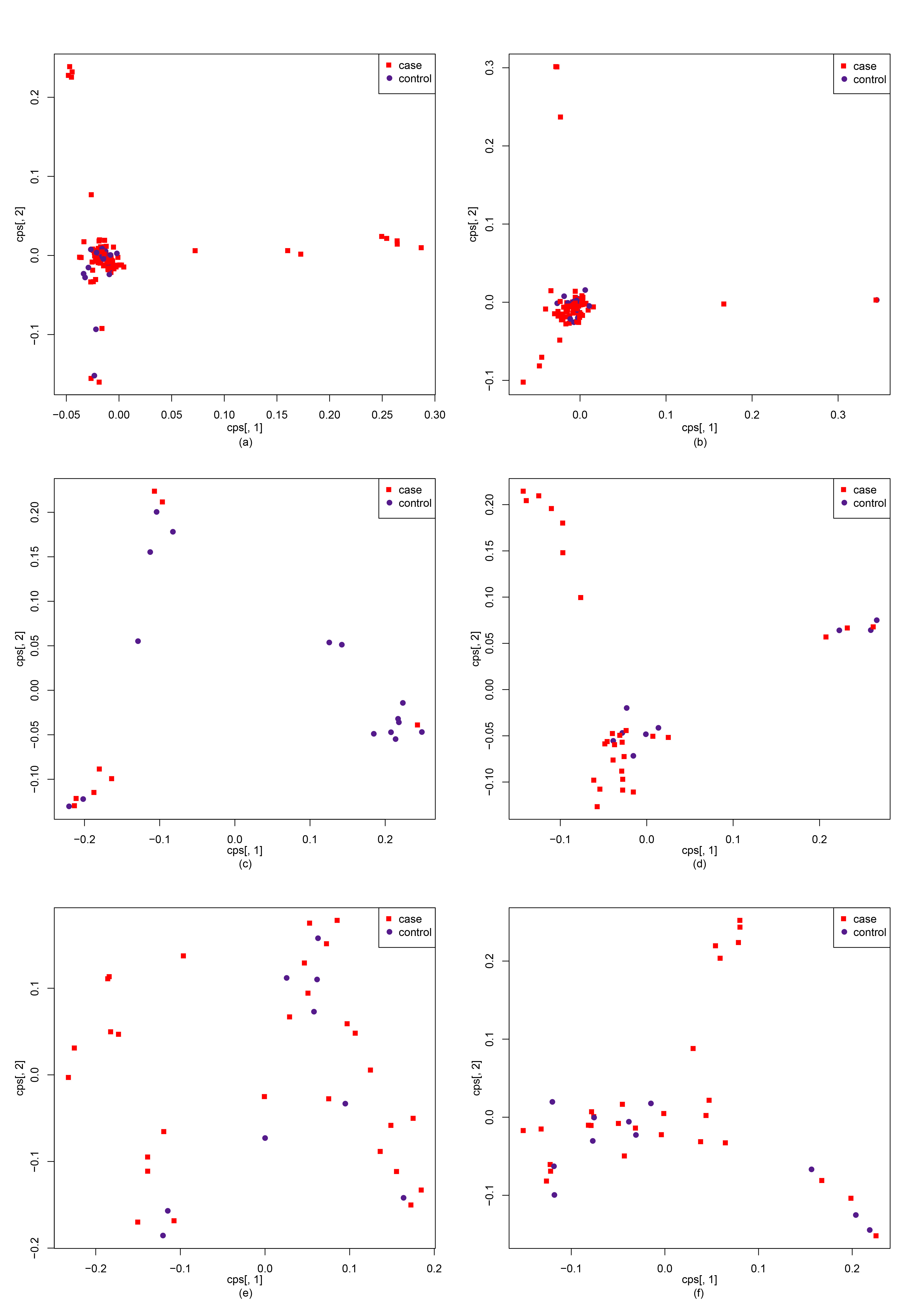

Supplement: FIGURE S3 — Multidimensional scaling (MDS) plots in (a) Wadi, (b) Hu, (c) Icelandic, (d) Finnish, (e) Romanov, and (f) Texel sheep. The red squares indicate animals from the case group (highly prolific ewes), and the purple dots represent animals in the control group (normally prolific ewes). [file Image_3.JPEG]

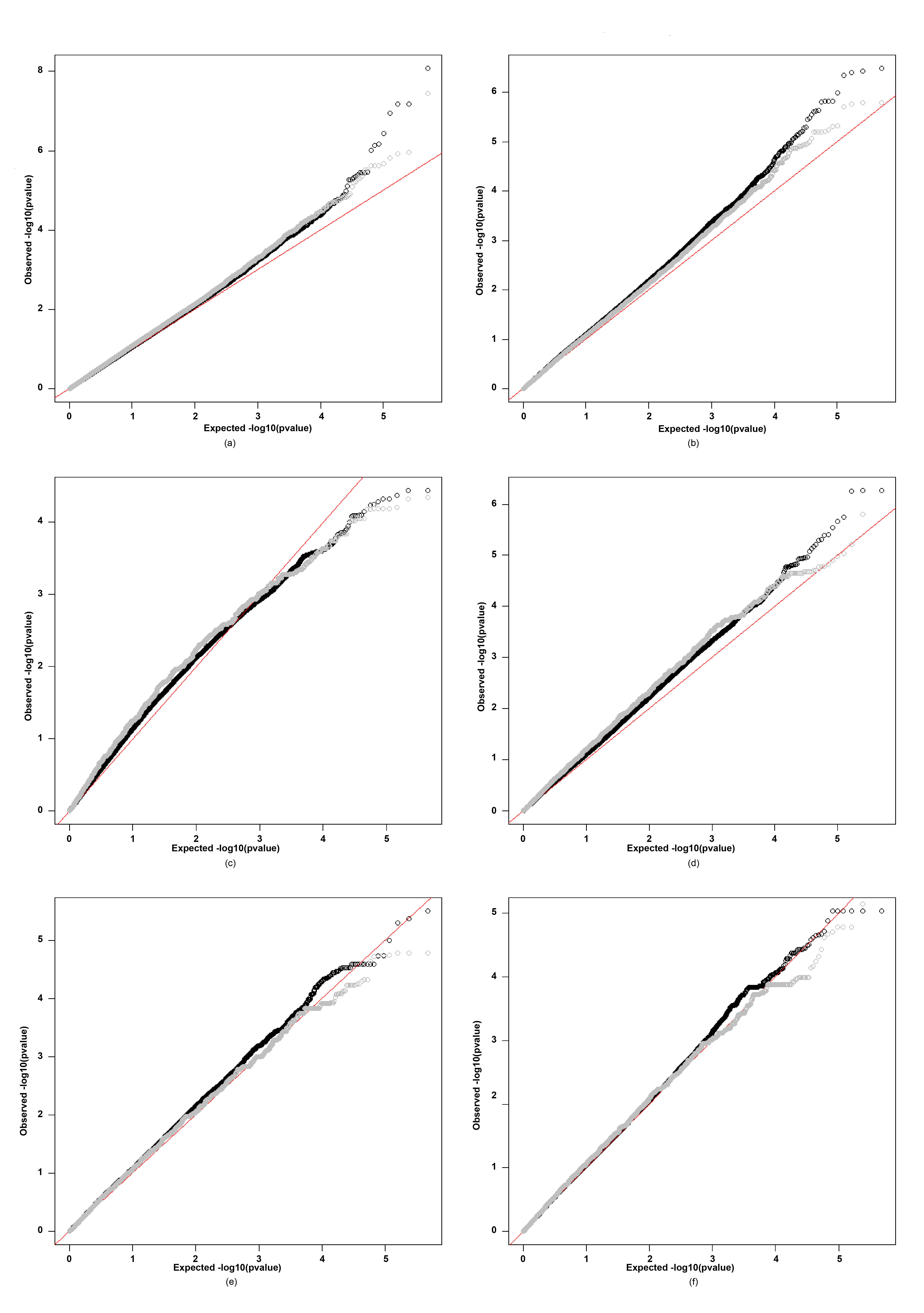

Supplement: FIGURE S4 — Q–Q (quantile–quantile) plots of GWAS in (a) Wadi, (b) Hu, (c) Icelandic, (d) Finnish, (e) Romanov, and (f) Texel sheep. Gray and black rings represent association statistics before and after correction for population stratification, respectively. [file Image_4.JPEG]
