# Supplementary material for: Genome-Wide Association Analyses Highlight the Potential for Different Genetic Mechanisms for Litter Size Among Sheep Breeds
Source: Front Genet. 2018 Apr 10;9:118. doi: 10.3389/fgene.2018.00118 (PMC5902979; doi:10.3389/fgene.2018.00118)
Supplement: Supplementary file 12 [file Data_Sheet_1.DOCX]

**Supplementary Information**

# Genome-wide association analyses highlights the potential for different genetic mechanisms for litter size among sheep breeds

# Song-Song Xu^1,2,^^†^, Lei Gao^3,4,†^, Xing-Long Xie^1,2^, Yan-Ling Ren^5^, Zhi-Qiang Shen^5^, Feng Wang^6^, Min Shen^3,4^, Emma Eyþórsdóttir^7^, Jón Hallsteinn Hallsson^7^, Tatyana Kiseleva^8^, Juha Kantanen^9^, Meng-Hua Li^1,^^*^

^1^CAS Key Laboratory of Animal Ecology and Conservation Biology, Institute of Zoology, Chinese Academy of Sciences (CAS), Beijing 100101, China

^2^University of Chinese Academy of Sciences (UCAS), Beijing 100049, China

^3^Institute of Animal Husbandry and Veterinary Medicine, Xinjiang Academy of Agricultural and Reclamation Sciences, Shihezi 832000, China

^4^State Key Laboratory of Sheep Genetic Improvement and Healthy Breeding, Xinjiang Academy of Agricultural and Reclamation Sciences, Shihezi 832000, China

^5^Shandong Binzhou Academy of Animal Science and Veterinary Medicine, Binzhou 256600, China

^6^Institute of Sheep and Goat Science, Nanjing Agricultural University, Nanjing 210095, China

^7^Faculty of Natural Resources and Environmental Sciences, Agricultural University of Iceland, Iceland

^8^All-Russian Research Institute for Farm Animal Genetics and Breeding, Russian Academy of Sciences, 55-a Moskovskoe Shosse, St. Petersburg-Pushkin 199601, Russia

^9^Green Technology, Natural Resources Institute Finland (Luke), Jokioinen 31600, Finland

^†^These authors contributed equally to this work.

**^*^**Corresponding author: M.-H. Li, Institute of Zoology, Chinese Academy of Sciences, Beichen West Road No. 1-5, Chaoyang District, Beijing 100101, China; E-mail: [menghua.li@ioz.ac.cn](mailto:menghua.li@ioz.ac.cn); Tel.: +86-10-64806336; Fax: +86-10-64806336

**MATERIALS AND METHODS**

Genotyping for all the samples was conducted using the Illumina Ovine Infinium HD SNP BeadChip and yielded a dataset of 606 006 SNPs. We implemented quality control of these SNPs using PLINK v1.07 software. The SNPs or individuals were excluded if they met any of the criteria: (1) no chromosomal or physical location, (2) call rate < 0.95, (3) missing genotype frequency > 0.05, and/or (4) minor allele frequency (MAF) < 0.05. SNPs were excluded from the analysis if a p-value of Fisher’s exact test for Hardy-Weinberg equilibrium less than 0.001. After filtering, a total of 406505 SNPs and 485 individuals were retained in the working dataset for the genome-wide association study (GWAS).

We performed the GWAS using a two-step approach via the general linear model and genome-wide efficient mixed-model analysis (GEMMA) across the six breeds. The first five principal components from a multi-dimensional scaling analysis were used as covariates to account for the biases caused by the population stratification in the GWAS. The models were detailed below:

(1) Residual calculation:

*y*=*µ* +*Tc* + *e* (model 1)

where *y* is the mean of litter size, *µ* is overall population mean, *c* is the first five principal components effect, *e* is the residual, *T* are the corresponding matrix vectors.

(2) Genome-wide efficient mixed-model analysis (GEMMA):

*e*=*µ**+ *Gk*+ *e** (model 2)

where *e* from the residual in model (1) is taken as the phenotype, *µ** is mean value of the population, *k* is the SNP effect, *G* is the genotype matrix vector, and *e** is the residual.

**RESULTS**

In the GWAS, we identified 17 SNPs (Table 2) at the chromosome- wise at the 5% level after the Bonferroni correction (Table 1). The annotation of the genes neighboring significant SNPs showed that no genes were associated with reproduction in human and livestock (Table 2).

**TABLE 1.** Bonferroni-corrected 5% chromosome-wise significance threshold in sheep.

| **Chromosome** | **5% chromosome-wide significance threshold** |
| --- | --- |
| 1 | 1.19732E-05 |
| 2 | 1.34156E-05 |
| 3 | 1.44634E-05 |
| 4 | 2.67237E-05 |
| 5 | 2.75028E-05 |
| 6 | 2.81057E-05 |
| 7 | 3.02663E-05 |
| 8 | 3.39674E-05 |
| 9 | 3.27869E-05 |
| 10 | 4.06835E-05 |
| 11 | 4.19463E-05 |
| 12 | 3.6049E-05 |
| 13 | 3.63901E-05 |
| 14 | 4.15628E-05 |
| 15 | 3.85802E-05 |
| 16 | 4.05515E-05 |
| 17 | 3.8432E-05 |
| 18 | 4.34783E-05 |
| 19 | 4.4405E-05 |
| 20 | 5.29101E-05 |
| 21 | 5.75374E-05 |
| 22 | 5.19751E-05 |
| 23 | 4.40141E-05 |
| 24 | 6.47668E-05 |
| 25 | 5.82751E-05 |
| 26 | 6.03865E-05 |
| 27 | 3.71747E-05 |

**TABLE 2.** Bonferroni-corrected genome-wide and chromosome-wise significant SNPs and their nearest gene based on the GWAS in the six sheep. The *p*_value corresponds to the corrected significance of GWAS after principle component adjustment. The symbol (/) denotes that no genes are located on upstream and downstream of the tested SNPs.

| **Chromosome** | **SNP** | **Position (bp)** | ***p*_value** | **Genes** |
| --- | --- | --- | --- | --- |
| 1 | *rs414097122* | 101017356 | 3.79075E-06 | *RPTN, TCHHL1* |
| 1 | [*rs421804262*](https://www.ncbi.nlm.nih.gov/projects/SNP/snp_ref.cgi?rs=421804262) | 213012691 | 9.82729E-06 | *TNFSF10, FNDC3B* |
| 3 | [*rs414883354*](https://www.ncbi.nlm.nih.gov/projects/SNP/snp_ref.cgi?rs=414883354) | 190445419 | 6.87487E-06 | *SOX5* |
| 7 | [*rs407090947*](https://www.ncbi.nlm.nih.gov/projects/SNP/snp_ref.cgi?rs=407090947) | 66997144 | 4.40251E-06 | */* |
| 7 | [*rs416107809*](https://www.ncbi.nlm.nih.gov/projects/SNP/snp_ref.cgi?rs=416107809) | 66996410 | 5.59763E-06 | */* |
| 7 | [*rs420563500*](https://www.ncbi.nlm.nih.gov/projects/SNP/snp_ref.cgi?rs=420563500) | 28748839 | 2.0192E-05 | */* |
| 8 | [*rs426132198*](https://www.ncbi.nlm.nih.gov/projects/SNP/snp_ref.cgi?rs=426132198) | 80603256 | 2.67054E-05 | *ZDHHC14* |
| 9 | [*rs400338438*](https://www.ncbi.nlm.nih.gov/projects/SNP/snp_ref.cgi?rs=400338438) | 91638261 | 2.50914E-05 | */* |
| 13 | *rs401592525* | 74413590 | 0.000011812 | *CDH22* |
| 14 | *rs400451936* | 56825967 | 1.61753E-05 | *PPP2R1A* |
| 16 | *rs414466013* | 30002131 | 2.81048E-05 | *MRPS30* |
| 17 | *rs422278203* | 18769443 | 1.82719E-05 | */* |
| 18 | *rs410531171* | 27261934 | 1.14478E-05 | *NSMCE3, FAM189A1, APBA2* |
| 20 | *rs412602669* | 41077012 | 1.44514E-05 | */* |
| 21 | *rs408253270* | 33212072 | 5.59613E-06 | *OPCML* |
| 24 | *rs411677584* | 37445078 | 3.00536E-05 | *NPTX2, BAIAP2L1, BRI3* |
| 27 | *rs421683185* | 95411539 | 3.49887E-05 | *PHF6, CCDC160, HPRT1* |
